# Neuroimaging Findings for the Overnight Consolidation of Learned Non-native Speech Sounds

**DOI:** 10.1162/nol_a_00157

**Published:** 2025-01-10

**Authors:** F. Sayako Earle, Peter J. Molfese, Emily B. Myers

**Affiliations:** Department of Communication Sciences and Disorders, University of Delaware, Newark, DE, USA; Center for Multimodal Neuroimaging, National Institute of Mental Health, Bethesda, MD, USA; Department of Speech, Language, and Hearing Sciences, University of Connecticut, Storrs, CT, USA

**Keywords:** consolidation, non-native speech, perceptual learning, sleep

## Abstract

Research over the past two decades has documented the importance of sleep to language learning. Sleep has been suggested to play a role in establishing new speech representations as well; however, the neural mechanisms corresponding to sleep-mediated effects on speech perception behavior are unknown. In this study, we trained monolingual English-speaking adults to perceive differences between the Hindi dental vs. retroflex speech contrast in the evening. We examined the blood oxygen level dependent signal using functional magnetic resonance imaging during perceptual tasks on both the trained talker and on an untrained talker shortly after training, and again the next morning. We also employed diffusion tensor imaging to determine if individual differences in white matter structure could predict variability in overnight consolidation. We found greater activity in cortical regions associated with language processing (e.g., left insula) on the second day. Fractional anisotropy values in the anterior thalamic radiation and the uncinate fasciculus were associated with the magnitude of overnight change in perceptual behavior on the generalization (untrained) talker, after controlling for differences in sleep duration and initial learning. Our findings suggest that speech-perceptual information is subject to an overnight transfer of information to the cortex. Moreover, neural structure appears to be linked to individual differences in efficiency of overnight consolidation.

## INTRODUCTION

When establishing a new speech category (e.g., in the case of learning the sound inventory of a new language), learners are confronted with a perceptual learning problem. Because of the many-to-few mapping between acoustic tokens and speech sound categories, speech category learning requires allowing one’s experience with acoustic-phonetic instances to define a distinct distribution within one’s perceptual space. This process entails discovering the acoustic cues that are relevant for defining the category, as well as the shape of that category for generalizing learned information to new category exemplars. During development, such representations are thought to reflect human sensitivity to the statistical properties of the native language input (Kuhl et al., 1992; Maye et al., 2002). Adults likewise track the statistical properties of speech sound categories (Kleinschmidt & Jaeger, 2015), but representational flexibility must be balanced against stability, to prevent new perceptual sensitivities from overriding the old. A potential means of maintaining this balance resides in the proposed mechanism for the offline consolidation of hippocampal information (*complementary learning systems* [CLS]; McClelland et al., 1995) in the slow integration of new sensory experiences with pre-existing category-level knowledge.

The CLS account of learning is a theoretical argument for the need for both temporary and long-term storage of memory. Broadly summarized, category formation presents a computational challenge. This problem is illustrated by the connectionist network simulations of learning conceptual categories (Rumelhart, 1990), wherein a category acquired through the convergence of shared features (e.g., both robins and canaries move, grow, and fly and therefore are exemplars of *bird*), and category building is undone by learning a nonprototypical exemplar of that category (e.g., penguins [which do not fly] are also birds). The proposed network solution is to gradually introduce nonprototypical exemplars alongside typical exemplars. Therefore, McClelland and colleagues hypothesized that the initial, rapid encoding of multimodal sensory input in the hippocampus is followed by the slow consolidation of this new information with pre-existing knowledge in the cortex. Importantly, this process must occur during an offline state (e.g., during sleep) when the hippocampus is not actively encoding new sensory experiences (Buzsáki, 1996; Pennartz et al., 2004).

Over the past three decades, there has been considerable empirical support for the role of sleep in the memory consolidation of episodic (hippocampal) with pre-existing (cortical) knowledge. In rats, pre-nap wake state experience is observed to be neurally “replayed” in the hippocampus during sleep (Wilson & McNaughton, 1994). Moreover, these replay events are temporally coordinated with cortical activity, suggesting a hippocampal-cortical dialogue taking place (Ji & Wilson, 2007; Qin et al., 1997). These events are associated with oscillatory spindle activity (Siapas & Wilson, 1998; Sirota et al., 2003), which are high-amplitude, high-frequency electrophysiological phenomena primarily observed during Stage 2 sleep (see De Gennaro & Ferrara, 2003, for review). Stage 2 sleep has been associated with declarative learning and post-sleep recall in humans (Clemens et al., 2005; Mednick et al., 2013; Tamminen et al., 2013; van der Helm et al., 2011). Such findings have provided empirical evidence that communication takes place between the hippocampus and the cortex during sleep and is important for declarative learning.

Compelling arguments for the role of sleep in the proposed memory transfer can also be found in the behavioral evidence for qualitative changes that occur to the memory trace after sleep. For example, it has been demonstrated that novel word forms (e.g., “cathedruke”) exert a lexical competition effect on pre-existing words (“cathedral”) only when a period of sleep had taken place between learning and assessment (Dumay & Gaskell, 2007; Dumay et al., 2004; Gaskell & Dumay, 2003). This is suggestive that a period of sleep is necessary for newly learned phonological strings to be integrated into the mental lexicon. Furthermore, a period of sleep (compared to a period of wake state) has been shown to promote the generalization of learned information to new situational contexts and to unfamiliar stimuli (Djonlagic et al., 2009; Friedrich et al., 2015; Gómez et al., 2006; Hupbach et al., 2009; Konrad et al., 2016; Lau et al., 2011). Such findings have been taken as evidence that episodic *gists*, such as situationally relevant sensory features, and/or rules that govern input structure, are abstracted away from the episodic (i.e., hippocampal) trace during sleep. These resulting abstractions, in turn, exert top-down influence over behavioral performance (see Diekelmann & Born, 2010, for review, and Gómez, 2011, for a more focused review on sleep’s role in generalization for language learning).

There is also a collection of functional magnetic resonance imaging (fMRI) evidence to support the narrative that sleep facilitates memory transfer. For example, Takashima et al. (2006) found that performance on a recognition memory task improved over the initial 24-hr period as a function of slow-wave sleep (SWS) duration. The same task was associated with decreases in hippocampal activity over the course of four fMRI sessions conducted over 90 days. This decrease in hippocampal activity corresponded to a gradual increase in ventral medial prefrontal regions, suggesting a neural reorganization. In the learning of novel phonological strings, Davis et al. (2009) found that cortical activity in regions associated with enhanced responses for pseudoword strings (left inferior frontal gyrus [LIFG] and left superior temporal gyrus [LSTG]) was similar in magnitude for unfamiliar novel phonological strings as compared to novel phonological strings learned on the day of the scan. By contrast, this activity was reduced for existing words as well as for novel phonological strings learned on the prior day. The authors also found elevated responses in the hippocampus to novel phonological strings prior to sleep. Together, it was suggested that the neural processing of novel phonological strings is qualitatively similar to existing words after, as compared with before, sleep. In yet another example, Durrant and colleagues (2013) found that probabilistic tone sequences elicited stronger striatal, and weaker parahippocampal, activity 24 hr after exposure, with respect to neural activity 30 min after exposure to the training stimuli. This increase in striatal activity was associated with the amount of SWS obtained during the intervening 24 hr as well as with the magnitude of improvement in behavioral performance. These studies share in common the interpretation that the involvement of the limbic system in performing newly learned tasks decreases after sleep, accompanying increased reliance on specialized networks.

In summary, there is convergent, cross-disciplinary evidence that initial learning undergoes neural transfer during sleep, and that this transfer process results in a qualitative change in the memory trace. This qualitative change can be described as going from episode-bound to an abstracted gist of the underlying principle(s) that bind together similar stimuli encountered across time. There is an obvious relevance for such a mechanism to the aggregation of disparate acoustic-phonetic encounters into cohesive speech sound categories. However, the behavioral observations of the role of sleep in speech-perceptual learning have been mixed, suggesting that the role of sleep in speech sound learning may not be directly analogous to previous studies on word learning.

### The Role of Sleep in Speech Sound Category Formation

Compared to learning in other domains, there is a relative paucity of studies that have directly investigated the role of offline consolidation in the perceptual learning of speech. Some perceptual learning tasks, such as lexically guided retuning of perceptual boundaries (Eisner & McQueen, 2006) or identification of syllables in noise (Roth et al., 2005), appear not to show performance changes that are specific to a post-training delay that contains sleep. In contrast, participants who were trained on identification of synthetic speech showed sleep-mediated effects on performance based on the time of day of training (Fenn et al., 2003) and the nature of the input (Fenn et al., 2013). Such disparate findings render the role of sleep in speech-perceptual learning unclear.

In contrast, the effects of sleep on speech sound learning may be different in learning tasks that introduce new perceptual categories to the phonological system. For example, previous work has examined the role of sleep on monolingual English speakers learning the dental (/d̪/) and retroflex (/ɖ/) contrast in Hindi, that perceptually assimilate to a single pre-existing category in English (/d/). It was observed that a period of post-training sleep had an either enhancing or degrading effect on discrimination between these targets, depending on when the participants were trained relative to sleep (Earle & Myers, 2015b). Furthermore, a period of sleep, but not a comparable period of wake state, facilitated improved perceptual accuracy on a set of tokens produced by an unfamiliar talker (Earle & Myers, 2015a). The latter effect of sleep-mediated generalization suggests that learned perceptual sensitivity may similarly be episode-bound prior to sleep, but then undergo a process of memory transfer whereby relevant features are abstracted.

The above findings are qualified by the observation that sleep-induced gains on perceptual discrimination following low-variability training are vulnerable to variability introduced during tests (e.g., Fuhrmeister & Myers, 2017). To illustrate, in training participants on a non-native lexical tone contrast in a single talker, but then testing on both the trained and an untrained talker, Qin and Zhang (2019) observed changes over time in identification performance, but not in discrimination performance. For the dental-retroflex contrast, discrimination performance has been found to improve after an evening training only when followed by a post-training test that utilized tokens produced by a single talker (Earle & Myers, 2015b; Earle et al., 2017). In contrast, when the post-training test included tokens by an untrained talker, discrimination performance was observed to decline overnight, while still facilitating cross-talker generalization in *identification* performance (Earle & Myers, 2015a).

It is unclear whether non-native phonetic category learning, like novel word learning, relies on a hippocampal-to-cortical transfer of information, as predicted by the CLS. In general, perception of native language speech sound categories relies on a cortical division of labor between the LIFG and the LSTG. The LSTG (as well as homologous structures in the right hemisphere) has been observed to maintain sensitivity to lower-level acoustic-phonetic detail, and shows tuning to native language category exemplars (Chang et al., 2010; Formisano et al., 2008; Liebenthal et al., 2005; Myers, 2007). In contrast, the LIFG appears to be involved in mapping graded acoustic phonetic information to a functional phonetic category, as well as resolution of phonetic ambiguity (Binder et al., 2004; Myers et al., 2009; Xie & Myers, 2018). In broad strokes, non-native category learning appears to recruit the same network: Behavioral metrics of successful category identification correspond to the recruitment of the LIFG (Callan et al., 2004; Golestani et al., 2007; Golestani & Zatorre, 2004; Myers & Swan, 2012), whereas the LSTG has been observed to develop training-induced changes to phonetic category structure (Guenther & Perkell, 2004; Leech et al., 2009; Yi et al., 2019). These findings suggest that learning non-native categories involves the retuning of neural sensitivities in this pre-existing network, and that non-native speech sound processing ultimately relies on the same network of structures that underlie native speech processing (for review, see Myers, 2014, and Myers & Fuhrmeister, 2023).

### Individual Differences in Overnight Consolidation

In the speech literature, as well as in other domains, the magnitude of behavioral change due to overnight consolidation is subject to both individual and group-level differences. In speech, intra-individual variability observed in just one night of overnight consolidation has been found to be associated with differences in language function such as reading skill (Williams & Earle, 2022). A similar association has been observed between reading ability and consolidation of recognition memory (Sengottuvel et al., 2020). In such cases, it has been argued that habitual differences in the consolidation of information (such as speech information), over time, may lead to substantial differences in the quality of linguistic representations. These differences in the quality of linguistic representations may, in turn, lead to differences in language skills. Aligning with this narrative, there is a growing literature documenting deficits in sleep-mediated consolidation in those with language and/or reading disorders (Ballan et al., 2023; Earle et al., 2018; Earle & Ullman, 2021; Malins et al., 2021; Reda et al., 2021; Sengottuvel et al., 2020; Smith et al., 2018). Sources of variability in overnight consolidation ability may therefore provide mechanistic insights into variability in language ability.

Research into the individual differences in consolidation of language knowledge have thus far examined functional sources of variability, such as oscillatory activity during initial memory formation (Heib et al., 2015) and sleep (Earle et al., 2018). By comparison, we know little about the differences in neural structures that modulate differences in consolidation of linguistic information, including in speech. Research from the broader consolidation literature, however, suggests that striatal white matter may play an important role. For example, it has been found that thalamocortical white matter microstructure predicts behavioral effects of motor memory consolidation (Vien et al., 2019). Vien and colleagues found that this association was mediated by sleep spindle activity, suggesting that individual differences in white matter structure may modulate potential for spindle generation. In other words, certain individuals may have the predisposition to be more efficient consolidators over others, given the same amount of sleep (Gaudreault et al., 2018). White matter structure may provide an index for such differences.

The purpose of the present study therefore was twofold. First, we examine the effect of sleep on neural activity for perception of a learned non-native contrast. We trained monolingual, English-speaking adults on the dental-retroflex contrast in the late evening. We then recorded participants’ neural activity while performing category identification using fMRI at two time points: immediately after training, and 12 hr later. Consistent with the CLS, we predicted that perceptual sensitivities would be episode-bound, and therefore task performance be reliant on the hippocampus, prior to a period of post-training sleep. In contrast, we predicted greater activation of cortical speech regions following a period of post-training sleep.

Our second research objective was to determine if white matter integrity would predict individual differences in the magnitude of overnight behavioral change, beyond differences in sleep duration. If hippocampal transfer underlies changes to speech-perceptual behavior overnight, and if thalamocortical white matter plays a role in sleep spindle generation, we expect to find associations between such tracts and overnight changes in perceptual behavior after controlling for sleep. We present our methods and results below.

## MATERIALS AND METHODS

### Participants

We recruited young adults between 18 and 24 years of age from the University of Connecticut community. We enrolled 17 participants; only 1 of these participants, however, was assigned male at birth so the sample was unbalanced. In consideration of sex-based differences in hippocampal learning (Koss & Frick, 2017; Piefke et al., 2005; Yagi & Galea, 2019), we excluded the data from the 1 male participant in the current analyses and present here the data on the 16 participants assigned female at birth. Participants were right-handed, monolingual speakers of American English, and all reported that they have no history of hearing or neurological deficits, nor a history of receiving any services for developmental language or reading disorders. All participants provided informed consent according to the institutional review board (IRB) guidelines and were screened for MRI safety with an IRB-approved risk assessment questionnaire. Participants were compensated at a rate of $10/hr for their time.

### Overview of Study Design

Participants were scheduled for two sessions on two consecutive days, such that Session 1 took place in the evening (either 6:00–8:00 p.m. or 7:00–9:00 p.m.), and Session 2 took place on the next morning (either 7:00–9:00 a.m. or 8:00–10:00 a.m.). This was done to ensure that all participants were scanned exactly 12 hr apart (7:00 p.m./7:00 a.m. or 8:00 p.m./8:00 a.m.), at roughly the same times of day.

**Figure 1.**
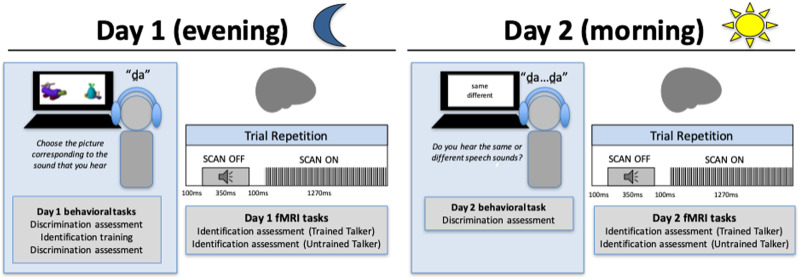
A graphical summary of the study procedures. Evening sessions were scheduled for a 2-hr block, such that participants spent the first hour engaged in the perceptual training program out of scanner. On Day 2, participants only completed a short (∼5 min) behavioral assessment prior to a second scan session, which was identical to the first.

During Session 1, participants first completed a behavioral training and assessment program on the dental-retroflex contrast, in a quiet room in a research laboratory adjoining the neuroimaging research suite. Immediately upon completion of the behavioral training, participants completed a 1-hr fMRI session during which they completed perceptual tasks on the learned non-native contrast in-scanner. During Session 2, participants completed a short behavioral assessment of the trained contrast, followed by a second scan session that was identical in procedural details to the first. See Figure 1 for graphical summary of study design.

At the end of the Day 1 session, participants were fit with an Actigraph wGT3X-BT accelerometer (Actigraph, https://theactigraph.com), programmed with participants’ biometric data and fitted on their non-dominant wrist. Actigraphs were set to collect data in 60-s epochs via a validated algorithm (Cain & Geremia, 2011) for collecting sleep versus wake activity over the 12-hr between-session interval to be entered as a covariate in our correlational analyses. This method of recording sleep/wake activity has been independently validated to agree with lab polysomnography at 85% for total sleep duration (Slater et al., 2015).

### Behavioral Procedure

Stimulus presentation and response recording was controlled using E-Prime 2.0 software (Psychology Software Tools, 2006). Auditory stimuli for the behavioral tasks were presented through SONY MDR-7506 Hi-Fi digital Sound Monitor headphones, at an average listening level of 70 dB sound pressure level.

#### Stimuli

Five unique tokens each of the target syllables (/ɖa/ - /d̪a/) were naturally spoken by two male native speakers of Hindi and recorded on Macintosh laptop in a sound-attenuated booth. Stimuli were cut to the onset of the burst, manipulated to match on duration (350 ms) and mean amplitude using PRAAT software (Boersma & Weenink, 2011). These stimuli had been used in a prior behavioral study in which the trained and untrained talkers were counterbalanced across learners (Earle & Myers, 2015a). In this prior study, we found behavioral evidence for consolidation using either token set during training; however, the learning rate was higher in one talker’s tokens over the other during the initial training. For the sake of our correlational analyses on individual differences, we sought to ensure that all participants experienced comparable opportunities to learn and to generalize across talkers. To this end, we designated the more difficult token set as the trained talker, and second talker’s token set as the untrained (generalization) talker, rather than counterbalancing across stimulus sets across participants. Auditory tokens were paired with two novel visual objects during training. (“Fribbles” stimulus images courtesy of Michael J. Tarr, Center for the Neural Basis of Cognition and Department of Psychology, Carnegie Mellon University, https://www.tarrlab.org.)

#### Perceptual training and behavioral assessments

##### Discrimination assessments.

Participants completed discrimination assessments at three time points. Immediately before training, immediately after training, and on the next day prior to the second scan session. Participants were played two tokens by the same talker in succession (1 s interstimulus interval) and asked to indicate whether the two tokens began with the same speech sound or with different speech sounds. Following eight practice trials with feedback, participants completed 128 trials of this task without feedback, in which 64 of the trials (32 trained/32 untrained) contained two exemplars of the same category (e.g., /ɖa_1_/ … /ɖa_2_/), and 64 of the trials (32 trained/32 untrained) contained tokens from different categories (e.g., /ɖa/ … /d̪a/).

##### Identification training.

Written instructions on the computer screen informed participants that they would be trained to perceive the difference between two speech sounds in a different language. Participants were played a familiarization sequence of trained talker’s token set (10 tokens, 5/dental, 5/retroflex), while the corresponding visual objects were presented on the screen. Afterward, both visual objects were presented on either side of the screen, and participants were asked to choose the picture corresponding to the sound that they heard. Participants completed 300 trials of this task, with a 2-min break after 150 trials. Participants were provided written feedback (correct/incorrect) after every trial.

### Neuroimaging Procedure

High-resolution 3D T1-weighted anatomical and functional MRI data were collected with a 3T Siemens Prisma scanner at the Brain Imaging Research Center at the University of Connecticut. Stimulus presentation and response recording was controlled using E-Prime 2.0. In-scanner auditory stimuli were presented using Avotec SilentScan 3300 with conformal headset (Avotec, Stuart, FL). In-scanner visual presentations were made using a BOLDscreen (Cambridge Research Systems, Rochester, UK). Responses were measured using a FORP 932 device with MR-compatible response buttons (Current Designs, Philadelphia, USA).

#### Data acquisition

Anatomical images were acquired using a multiecho magnetization prepared rapid acquisition gradient sequence (ME-MPRAGE; echo time [TE] 2.98 ms, inversion time [TI] 900 ms, 1 mm^3^ isotropic voxels, 248 × 256 matrix, 176 slices). Functional data (30 axial slices of 3.125 mm thick echo planar images [EPI]) were obtained in interleaved ascending order (94 × 94 matrix, 224-mm^3^ field of view [FOV], flip angle = 90°), in 4 runs of 121 trial repetitions (TRs; acquisition time 1,820 ms). Data were collected using a sparse-sampling design (Edmister et al., 1999), in which auditory stimuli were presented within brief (550 ms) gaps inserted between scans, resulting in a final effective TR of 2,390 ms. This design has been widely used to study neural responses to fine-grained distinctions among auditory stimuli (e.g., Liebenthal et al., 2010; Myers et al., 2009). Stimulus presentation was randomized to occur every one to seven TRs (average of five TRs).

Diffusion tensor images (DTI) were acquired with resolution 2 mm × 2 mm × 3 mm with FOV 128 × 128 along 40 axial slices; TR of 9,000 ms, TE of 94 ms. Two separate phase encoding acquisitions (AP, PA) were collected with 30 (bval = 1,000) diffusion directions each, as well as 4 b0-weighted images, rendering 68 total volumes for processing. To aid in later processing of DTI data, we also acquired a T2-space image with sagittal acquisition parameters 256 × 256 mm FOV, TR 3,200, TE 564 ms, 160 slices.

#### Identification task during scanning

The in-scanner task resembled the training (identification) task except that no feedback was provided. The two visual objects were presented on the screen while auditory stimuli were presented via MR-compatible circumaural headphones. Participants were asked to indicate which picture corresponded with each auditory token via button press. Each run of 121 TRs contained 40 task trials, arranged to occur pseudo-randomly every 1 to 5 TRs (average of 3 TRs between trials). Only the trained talker’s tokens were played during the first two runs, followed by the untrained talker’s tokens for runs 3 and 4, for a total of 80 identification trials (40 dental/40 retroflex) per talker.

## RESULTS

### Behavioral Performance

In-scanner measures of behavioral performance for the majority of our participants were lost due to error in equipment settings for the response box. Behavioral analyses therefore involve perceptual performance only on tasks performed outside of the scanner. Accuracy in the discrimination task was transformed to *d*′ scores, defined as the *z*-score of the hit rate (proportion of different trials correctly identified) minus the *z*-score false alarm rate (proportion of same trials incorrectly identified as different; Macmillan & Creelman, 2005).

**Figure 2.**
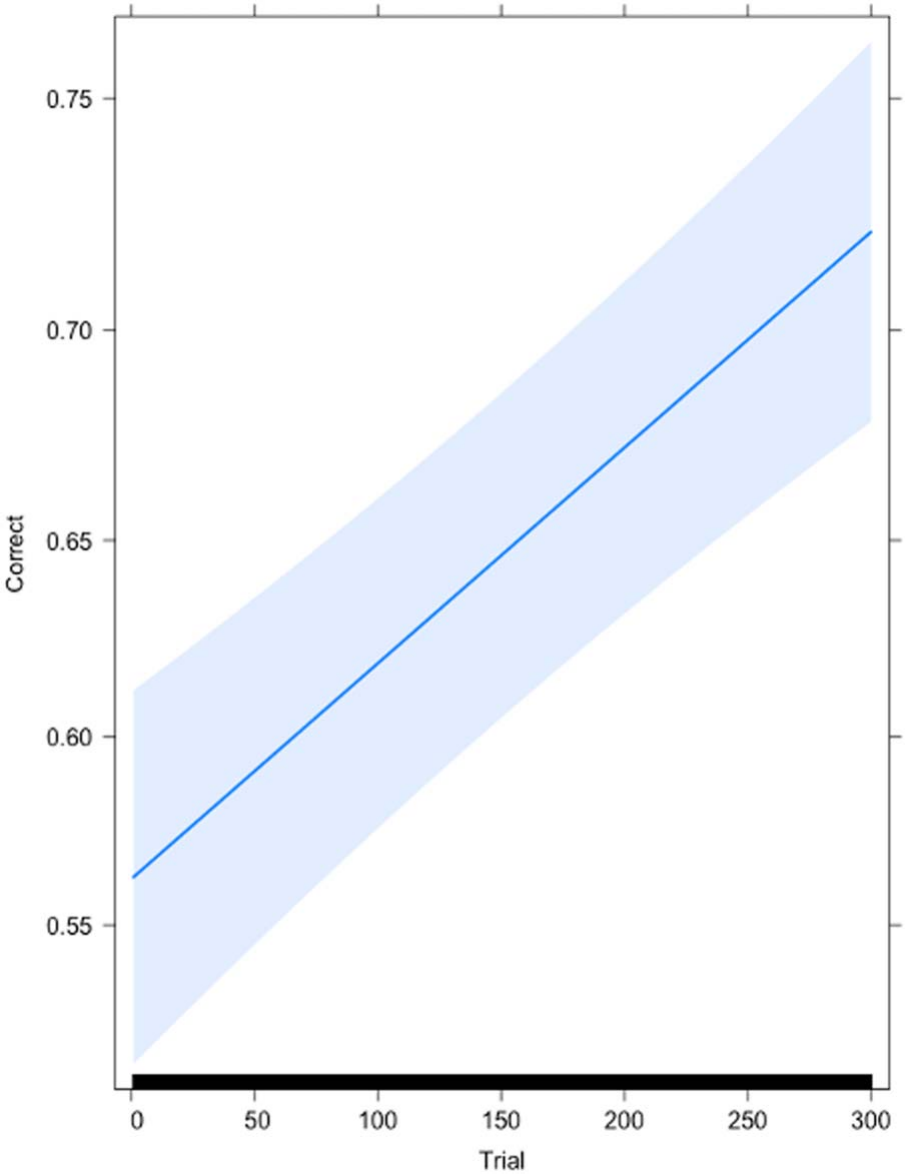
Effect plot for Accuracy by Trial number during identification training. The probability of a correct response on any given trial (expressed as percentage on the *y* axis), is near chance levels at early trials (*x* axis), but approximates 75% by the end of training. Error bars denote 95% confidence interval of the effect estimate.

In order to ensure that participants learned the contrast prior to the scan sessions, we fitted a logistic generalized mixed-effects model over the trial-by-trial accuracy during identification training, with trial number and baseline performance entered as fixed effects, and participant as a random factor. The resultant model (Akaike information criterion [AIC] = 6,141.4, Bayesian information criterion [BIC] = 6,167.3, Logliklihood = −3,066.7, r2m = 0.01, r2c = 0.05) contained a significant main effect of Trial, *β* = 0.002, *SE* < 0.001, *p* < 0.001, r2*β* = 0.009. Baseline performance was not significant (*p* = 0.405). This indicated that participants were more likely to be accurate on trials that took place at the end of training relative to the beginning. See Figure 2 for a graphical depiction of learning rate.

As we had lost the data on the identification task in-scanner, our means of tracking perceptual change across time was limited to performance measures on the out-of-scanner discrimination task only. We note that, as discussed above, consolidation effects on discrimination performance have varied across different stimulus sets used during post-training tests. In particular, the use of an untrained talker’s tokens during testing (such as in the current work) has been observed to result in degraded discrimination performance on the trained talker’s tokens following an overnight delay, even while enhancing identification performance on the same tokens (Earle & Myers, 2015a; Qin & Zhang, 2019). Thus, discrimination performance on the trained talker may not be the best indicator of consolidation in the current context.

**Figure 3.**
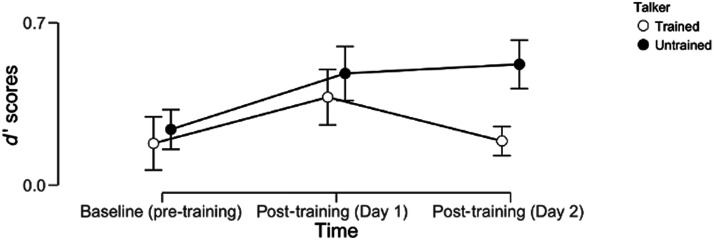
Descriptive plot of behavioral performance on the out-of scanner discrimination task. Error bars denote standard errors of the mean.

Nevertheless, to determine changes to perceptual accuracy, we conducted a repeated measures analysis of variance (ANOVA) on the Discrimination scores on two levels of Talker (Trained/Untrained) and three levels of Time (Pre-training, Post-training [Day 1], and Post-training [Day 2]). A Mauchly’s test was conducted to ensure that data met assumptions of sphericity. This analysis revealed a significant main effect of Time (*F*_2,30_ = 4.83, *p* = 0.015, *η*^2^ = 0.060) but no main effect of Talker (*F*_1,15_ = 1.70, *p* = 0.212, *η*^2^ = 0.050) nor an interaction between Time and Talker (*F*_2,30_ = 0.998, *p* = 0.381, *η*^2^ = 0.016). The source of the Time main effect was determined to be driven primarily by difference in performance between baseline and immediate post-training performance (*t*(15) = −3.053, *p* = 0.014, *d* = −0.483), with nonsignificant changes in performance observed overnight (*t*(15) = 1.021, *p* = 0.947, *d* = 0.316; Bonferroni correction applied). See Figure 3 for graphical depiction.

### Overnight Changes in Neural Activation

Using AFNI (Cox, 1996), data were motion-corrected using a six-parameter rigid-body transform, aligned to each person’s high-resolution anatomical (ME-MPRAGE) dataset, and transformed to Talairach space (Colin27; Talairach & Tournoux, 1988). The datasets were spatially smoothed with a 6-mm Gaussian kernel. Motion and signal fluctuation outliers were removed, and stimulus onset times were convolved with a stereotypic gamma hemodynamic function to model the time course for each token type (retroflex/dental) and Talker. These vectors were regressed with six additional movement parameters as covariates to determine fit coefficients for each token type and Talker, by Participant by Day.

These fit coefficients were then submitted to a 2 × 2 repeated measures analysis of variance (ANOVA) using 3dMVM (AFNI), with Talker (Trained vs. Untrained) and Time (Day 1 vs. Day 2) as the within-subjects factors. To determine the appropriate thresholds for cluster-level corrections, a group mask was first created. Then, the spatial autocorrelation function of 3dClustSim (AFNI) was applied on the group mask. This method is consistent with current standards for second-level correction (Cox et al., 2017; Eklund et al., 2016). Based on these results, we applied a voxel level threshold of 0.01 and 222 contiguous voxels, to maintain a cluster level correction of alpha < 0.05.

We observed significant main effects of Day, Talker, and a significant interaction between Day and Talker. The main effect of Day was driven by greater activation observed in the left insula on Day 2 relative to Day 1. The main effect of Talker was driven by greater activity in regions usually unassociated with speech processing listening to the Untrained Talker (left orbital gyrus, left precuneus), and greater activity in regions associated with speech by the Trained Talker (left postcentral gyrus, right supramarginal gyrus). The Day by Talker interaction was observed in the left calcarine gyrus, driven by greater activity for the Untrained Talker on Day 2. The list of significant effects and their corresponding regions is found in Table 1. In addition, as we had hypothesized a greater activation in the hippocampus on Day 1, we examined the activity in the bilateral hippocampus. In doing so, we observed greater activity on Day 1 relative to Day 2 in the left hippocampus, in a small cluster that did not meet our threshold (33 voxels).

**Table 1.** Main effects and interactions from 2 × 2 repeated measures ANOVA

	Maximum intensity coordinates			# voxels	*t* value
	*x*	*y*	*z*		
Day					
Day 2 > Day 1					
Left insula	38.7	1.7	12.4	223	3.58
Talker					
Untrained > Trained					
Left orbital gyrus	1.4	−46.3	0.5	775	4.04
Left precuneus	7.9	50.3	26.7	447	3.59
Untrained < Trained					
Left postcentral gyrus	43.6	31.3	43.9	1,115	3.55
Right supramarginal gyrus	−47.2	31.2	36.1	572	3.65
Day × Talker					
Day 1, Untrained < Trained					
Left calcarine gyrus	−0.9	76.9	7.9	293	2.54
Day 2, Untrained > Trained					
Left calcarine gyrus	−0.9	76.9	7.9	293	1.59

### White Matter Structure as Predictors of Overnight Change

Diffusion data for each phase encoding (AP and PA) were preprocessed in TORTOISE 3.0 (Irfanoglu et al., 2017; Pierpaoli et al., 2010), which provides corrections for motion, eddy distortions, B0 and field distortions using a single interpolation. The two-phase encodings were then combined using Diffeomorphic Registration for Blip-Up blip-Down Diffusion Imaging (DR-BUDDI; Irfanoglu et al., 2015) to properly combine the blip-up and blip-down data. Data were then processed in Freesurfer (Fischl, 2012) and Tracts Constrained by Underlying Anatomy (TRACULA; Kreilkamp et al., 2017; Yendiki et al., 2011), yielding tract statistics on 18 total tracts. For these, we examined the fractional anisotropy (FA) values of each tract in relation to out-of-scanner behavioral measures.

As an initial pass at examining relationships between white matter structures and behavioral performance, we first compared overnight changes in behavior (Day 2 − Day 1 discrimination performance) for the Trained and Untrained Talkers separately to FA values from each of the 18 tracts reconstructed by TRACULA. After applying the Holms-Bonferroni correction for multiple correlations, we found significant associations between overnight changes in discrimination ability for the Untrained Talker, and the bilateral anterior thalamic radiation (ATR), as well as the bilateral uncinate (UNC). See correlation matrix in Table 2.

**Table 2.** Correlation matrix of diffusion tensor imaging tracts and overnight change in behavior

Talker	Hemisphere	ATR	CAB	CCG	CST	ILF	SLFP	SLFT	UNC	fmajor	fminor
Trained	Left	−0.03	−0.01	0.06	−0.11	−0.06	0.4	0.35	−0.05	0.09	0.25
	Right	−0.08	−0.18	0.01	−0.21	−0.38	0.04	0.01	−0.12		
Untrained	Left	0.76*	0.45*	0.43	0.25	0.48	0.57	0.62	0.63*	−0.18	0.29
	Right	0.76*	0.39	0.58	0.19	0.51	0.53	0.58	0.66*		

*Note*. Pearson’s *R* values for correlations between overnight change in behavior and white matter tracts.

* Denotes statistical significance at the 0.05 level following Holms-Bonferroni correction for family-wise error. Tracts reconstructed by TRACULA (Kreilkamp et al., 2017) include anterior thalamic radiation (ATR), cingulum-angular bundle (CAB), cingulum–cingulate bundle (CCG), inferior longitudinal fasciculus (ILF), superior longitudinal fasciculus–parietal bundle (SLFP), the uncinate (UNC), and the forceps major (fmajor) and minor (fminor).

**Figure 4.**
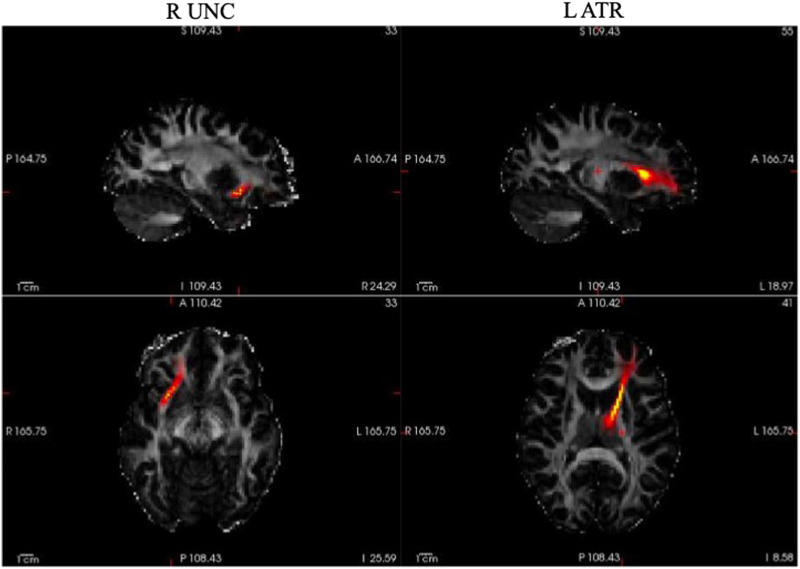
Visualization of the uncinate (UNC) and the anterior thalamic radiation (ATR), as the two DTI tracts identified to be closely associated with consolidation behavior. The images above are in radiological view (i.e., left on right).

We chose to examine further the correlations between overnight changes to discrimination behavior in the untrained talker and ATR and UNC, as these were the tracts observed with strong bilateral associations with behavior (Figure 4). Specifically, we wanted to rule out the possibility that these correlations were epiphenomenal to relationships between these tracts with sleep duration, or with initial learning ability. To this end, we fit a set of linear mixed-effects models (VanLeeuwen et al., 1996) on the overnight changes to discrimination performance in R (Version 4.1.3; R Core Team, 2022).

For these models, wrist actigraphy provided measures of total sleep duration for all but two of our participants, whose data supplied relative activity levels for the between-session interval. Activity levels during the day were used to impute total sleep duration for these participants via the Multivariate Imputation by Chained Equation package in R (mice; van Buuren & Groothuis-Oudshoorn, 2011). We deviation-coded Talker (Trained −0.5 vs. Untrained 0.5), and computed average FA values across hemispheres for the ATR and UNC.

The first mixed effects model contained an interaction term between Talker and ATR, Learning Rate (defined as the total number of correct trials during training), baseline Discrimination, and Sleep duration, with by-participant intercepts modeled as a random factor. The resultant model AIC = 39.5, BIC = 52.7, Logliklihood = −10.8, r2m = 0.45, r2c = 0.45 contained a significant main effect of Talker, *β* = −9.10, *SE* = 2.71, *p* = 0.002, r2*β* = 0.286, a significant main effect of ATR, *β* = 7.75, *SE* = 3.20, *p* = 0.021, r2*β* = 0.173, and a significant interaction between Talker and ATR, *β* = 21.27, *SE* = 6.19, *p* = 0.002, r2*β* = 0.297. Post-hoc regressions determined this interaction to be driven by a significant relationship between the ATR and overnight changes in discrimination ability for the Untrained talker, *β* = 19.02, *SE* = 3.75, *p* < 0.001, but not for the Trained talker, *β* = −3.35, *SE* = 6.32, *p* = 0.605.

The second mixed effects model contained an interaction term between Talker and UNC, Learning rate and Sleep duration, and Baseline discrimination, with by-Participant intercepts modeled as a random factor. The resultant model AIC = 43.4, BIC = 56.6, Logliklihood = −12.7, r2m = 0.34, r2c = 0.34 contained a significant main effect of Talker, *β* = −8.53, *SE* = 2.88, *p* = 0.006, r2*β* = 0.239 and a significant interaction between Talker and UNC, *β* = 19.96, *SE* = 6.55, *p* = 0.005, r2*β* = 0.249. Post-hoc regressions determined this interaction to be driven by a significant relationship between the UNC and overnight changes in discrimination ability for the Untrained talker, *β* = 15.85, *SE* = 4.52, *p* = 0.004, but not for the Trained talker, *β* = −4.80, *SE* = 6.67, *p* = 0.796.

**Figure 5.**
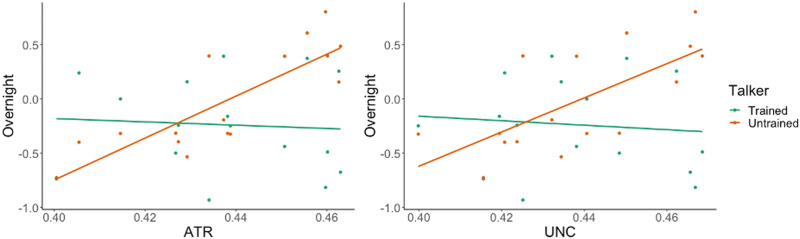
Scatterplots of overnight changes in perceptual ability (difference in *d*′) by Talker (*y* axis) against average fractional anisotropy values for the anterior thalamic radiation and uncinate (*x* axis).

In summary, both the ATR and UNC emerged as significant predictors of the magnitude of overnight changes in discrimination accuracy for the Untrained talker, but not for the Trained talker, after controlling for learning rate and sleep duration. See Figure 5 for a graphical depiction of these relationships.

## DISCUSSION

The current study examined the neural indexes of sleep-mediated changes associated with speech-perceptual learning, as well as individual differences in neural structure associated with efficient consolidation. We first examined overnight changes in neural activation in the perception of a trained non-native contrast. We then examined the relationship between white matter structure and the magnitude of overnight consolidation for speech.

Notably, activation varied by virtue of both the talker (whether it was the trained or untrained talker) and the interaction between talker and testing day. Activation for the trained talker was greater in the left postcentral gyrus and right supramarginal gyrus. The right posterior temporal and temporoparietal cortex has been implicated for processing talker identity (e.g., Kriegstein & Giraud, 2004; Myers & Theodore, 2017; see Luthra, 2021, for review). Greater activation for the untrained talker was observed in the left orbital gyrus and left precuneus—areas that do not have an obvious relationship with talker processing. The involvement of the visual cortex in the interaction between talker and testing day may reflect greater visual analysis of the novel objects for the purpose of mapping the tokens produced by the untrained talker on the second day.

Of interest to our primary research question, the main effect of Day observed in the left insula indicates that language-related cortex is recruited as the speech categories are solidified. Although the role of the insula in language has been the subject of much debate, evidence from cortical recording suggests a role for the posterior insula in speech planning, particularly internal speech (Woolnough et al., 2019). Speculatively, emergence of activation in the insula on the second day may reflect recruitment of speech motor systems to differentiate the perceptual categories. Notably, while activation increased in the insula on the second day, there is a hint that the same tasks may be more reliant on the hippocampus on Day 1. Together, these findings may support the narrative that speech-perceptual information undergoes a hippocampus-to-cortex transfer of information overnight. These findings are consistent with patterns of neural activation observed for the integration of novel forms into the mental lexicon overnight (Davis et al., 2009). Thus, the overnight consolidation effects observed on speech-perceptual behavior (Earle & Myers, 2015a, 2015b; Earle & Qi, 2022; Fenn et al., 2003, 2013) may parallel the neural time course of word learning, but at the segmental tier.

We also found the FA values of two anterior tracts to be positively associated with overnight changes in the perception of the untrained talker, beyond differences in sleep duration and in training-induced learning. As task performance for the untrained talker is less likely to rely on episodic features encountered during training, this condition may reflect the degree to which a participant successfully extracts a generalizable set of features away from the training stimuli. This line of reasoning suggests that the integrity of the ATR and UNC predict the degree to which acoustic features generalize overnight. This finding is consistent with prior observations in motor learning (Vien et al., 2019), and hints that differences in anterior white matter may lead to differences in relative efficiency in consolidation. This finding may have broader implications for the mechanisms underlying learning disability, as poor consolidation of speech has been observed in developmental language disorder (Earle et al., 2018; Malins et al., 2021) and with poor decoding ability (Williams & Earle, 2022).

The role of thalamocortical radiations in control over cortical arousal, consciousness, and sleep-wake regulation has been extensively studied (George & Das, 2020). While the roles of UNC are less clear, some have proposed that the UNC is involved in modulating temporal lobe-based mnemonic associations, and in the acquisition and expression of memory in the medial temporal lobe (Von Der Heide et al., 2013). Alterations in the ATR and UNC have been linked to various psychiatric disorders that affect sleep (e.g., bipolar disorder, major depression, schizophrenia; Deng et al., 2018; Lai & Wu, 2014; McIntosh et al., 2008), as well as to different neurodevelopmental disorders (e.g., autism, Cheon et al., 2011; dyscalculia, Ayyıldız et al., 2023; McCaskey et al., 2020). Relevant to the learning and retention of speech, the developmental trajectory of the internal capsule (inclusive of the ATR) has been found to be reduced in those with developmental language disorder (Lee et al., 2020), whereas the integrity of the UNC is associated with dyslexia (Mohammad et al., 2022; Zhao et al., 2022). Taken together with current findings, the association between white matter integrity and consolidation of speech may play a mechanistic role in poor speech sound representations, although the directionality of these relationships are as yet unclear.

The current study does have some important limitations to acknowledge. First, our sample consisted of young adults assigned female at birth only, and thus it is possible that our observations may not generalize to individuals assigned male at birth. Sex differences in hippocampal function is a growing area of research (Koss & Frick, 2017). Future research may reveal sex-related differences on the relative role of the hippocampus during speech-perceptual learning, which may affect downstream consolidation effects. We also acknowledge that our decision to employ one talker consistently for training and another to assess generalization may potentially introduce talker-specific effects into our findings. Future studies will employ additional talkers to assess generalization, such that the untrained status of a talker is not tied to a single talker’s idiosyncrasy. Furthermore, our brain-behavioral relationships were examined with respect to speech discrimination performance only, due to loss of behavioral data on the identification task. While the lack of perceptual gains observed in the discrimination task is consistent with other studies that have used tokens from multiple talkers during the post-training test (Earle & Myers, 2015a; Qin & Zhang, 2019), we can only speculate here that the changes in performance on the untrained talker is attributable to consolidation. Additionally, as discrimination and identification may require different skills and may pattern differently over time (e.g., Earle & Myers, 2015b), our team is currently engaged in the effort to track consolidation-induced changes to both speech discrimination and speech identification behavior. Finally, our overnight design implies a circadian difference between the two fMRI scan sessions, and thus we are unable to rule out time-of-day effects in the observed differences in behavior.

Despite these limitations, the current findings have important implications for perceptual learning of speech. First, there is likely to be qualitative differences in the speech-perceptual performance following a period of post-training sleep, reflecting neural changes to the speech representation. This may affect the interpretation of research findings on speech-perceptual learning that take place over multiple days. Second, there may be anatomical differences beyond differences in sleep hygiene alone to account for differences in consolidation. This may have important implications for learning disabilities associated with relatively weak overnight consolidation (Earle et al., 2018; Earle & Ullman, 2021). These are directions that we intend to pursue in the future.

## ACKNOWLEDGMENTS

The authors would like to thank Elise Medeiros for facilitating the off-hour scans, and Stephanie Del Tufo, who was consulted in the initial design of the fMRI task. FSE was supported by an ASH Foundation scholarship and the Fund for Innovation in Science Education at the University of Connecticut. The content is the responsibility of the authors and does not necessarily represent official views of our funding sources.

## FUNDING INFORMATION

Emily B. Myers, National Institute on Deafness and Other Communication Disorders (https://dx.doi.org/10.13039/100000055), Award ID: R01DC013064. Emily B. Myers, National Science Foundation, Award ID: BCS 1554810. F. Sayako Earle, National Institute on Deafness and Other Communication Disorders (https://dx.doi.org/10.13039/100000055), Award ID: F31 DC014194. Eunice Kennedy Shriver National Institute of Child Health and Human Development (https://dx.doi.org/10.13039/100009633), Award ID: P01 HD001994. F. Sayako Earle, American Speech-Language-Hearing Foundation (https://dx.doi.org/10.13039/100002607), Award ID: New Century Scholars Doctoral Scholarship.

## AUTHOR CONTRIBUTIONS

**F. Sayako Earle**: Conceptualization: Equal; Formal analysis: Supporting; Funding acquisition: Equal; Investigation: Lead; Methodology: Lead; Project administration: Lead; Visualization: Equal; Writing – original draft: Lead. **Peter J. Molfese**: Formal analysis: Lead; Investigation: Supporting; Methodology: Supporting; Visualization: Equal; Writing – review & editing: Supporting. **Emily B. Myers**: Conceptualization: Equal; Funding acquisition: Equal; Investigation: Supporting; Methodology: Supporting; Project administration: Supporting; Resources: Lead; Supervision: Lead; Writing – original draft: Supporting; Writing – review & editing: Supporting.

## DATA AND CODE AVAILABILITY STATEMENT

Deidentified data presented in this manuscript and the code used for analyses are accessible through the Open Science Framework or GitHub. The behavioral data, r code used for analyses, and anatomical and functional neuroimaging datasets are accessible through this link: https://osf.io/mtc32/. The DTI dataset is available here: https://osf.io/yvkcu/. The code used for processing and analyzing the neuroimaging data is available here: https://github.com/pmolfese/EarleNBL2024_MTOPL.

## TECHNICAL TERMS

Consolidation: Refers to the processes by which a new memory trace is stabilized/converted into long-term memory.
Phonetic: Refers to features of speech that may not necessarily signal a change in speech sound category.
